# VEGETABLE CELLULOSE NANOFIBER DRESSING AIDS IN THE HEALING PROCESS OF THIRD-DEGREE BURNS? STUDY ON RATS

**DOI:** 10.1590/0102-672020210002e1586

**Published:** 2021-10-18

**Authors:** Milka Lie TAKEJIMA, Maria Angelica Baron MAGALHÃES, Jurandir Marcondes RIBAS, Fernando Issamu TABUSHI, Carlos Cesar Bof BUFON, Thayline Mylena Santana CAMARGO, Isabela Calixto MALUF, Osvaldo MALAFAIA

**Affiliations:** 1 Mackenzie Evangelical Faculty of Paraná, Curitiba, PR, Brazil; 2 University Evangelical Mackenzie Hospital, Curitiba, PR, Brazil

**Keywords:** Burns, Wound Healing, Cellulose, Nanotechnology, Nanostructures, Wounds and Injuries, Queimaduras, Cicatrização, Celulose, Nanotecnologia, Nanoestruturas, Ferimentos e lesões

## Abstract

***Background*:**

The treatment of 3^rd^ degree burns represents a major medical challenge. Pinus vegetable cellulose is a biomaterial with characteristic similar to bacterial cellulose.

***Aim*:**

To evaluate the safety of cellulose membrane (*Pinus sp*) in the treatment of 3^rd^ burns in rats and to compare its effectiveness with the bacterial membrane already on the market.

***Method*:**

Thirty-three Wistar rats were beaten with a 3^rd^ degree burn on back skin by applying water at 98º C for 30 s. Then, they were divided into three groups (n=11): group 1 - simple dressing with gauze; group 2 - dressing with bacterial cellulose membrane; and group 3 - dressing with vegetable cellulose membrane. The animals were maintained for 15 days to check the general clinical status, macroscopic aspect, contraction of the wounds and microscopic analysis for the degree of healing and collagenization.

***Results*:**

They were clinically well during the experiment. During the removal of the dressing, there was bleeding in the wound of the control group, unlike the groups treated with cellulose membranes, which protected the bed from injury. The macroscopic evaluation showed a greater contraction of the wounds treated with the membranes in relation to the control. A microscopic analysis revealed that most of the wounds were in advanced healing degree with predominance of mature collagen in all groups.

**Conclusion:**

*Pinus sp* cellulose membrane showed efficacy similar to that of the bacterial membrane in the treatment of 3^rd^ degree burns.

## RESUMO

***Racional*:**

O tratamento das queimaduras de 3˚ grau representa grande desafio na área médica. A celulose vegetal de pinus é biomaterial com características semelhantes às da celulose bacteriana**.**

***Objetivo*:**

Avaliar a segurança da membrana de celulose vegetal (*Pinus sp*) no tratamento de queimaduras de terceiro grau em ratos e comparar sua eficácia com a da membrana bacteriana já comercializada. ***Método*:** Trinta e três ratos Wistar foram submetidos à queimadura de 3º grau na pele do dorso mediante aplicação de água a 98º C durante 30 s. Em seguida, foram distribuídos em três grupos (n=11): grupo 1 - curativo simples com gaze; grupo 2 - curativo com membrana de celulose bacteriana; e grupo 3 - curativo com membrana de celulose vegetal . Os animais foram avaliados durante 15 dias para verificar o estado clínico geral, aspecto macroscópico, contração das feridas e análise microscópica pelo grau de cicatrização e colagenizacao.

***Resultados*:**

Permaneceram clinicamente bem durante o experimento. Durante a retirada do curativo houve sangramento na ferida do grupo controle, diferentemente dos grupos tratados com as membranas de celulose, que protegeram o leito da lesão. A análise microscópica mostrou que a maioria das feridas apresentava-se em grau avançado de cicatrização, com predomínio de colágeno maduro em todos os grupos. Houve maior contração das feridas tratadas com as membranas em relação ao grupo controle.

**Conclus:**

*ão*: A membrana de celulose de *Pinus sp* apresentou eficácia semelhante à da membrana bacteriana no tratamento de queimaduras de 3˚ grau.

## INTRODUCTION

Burns are considered a major public health problem that affects all age groups and social classes^4^. According to the World Health Organization^30^, they are responsible for approximately 180,000 deaths per year worldwide. In Brazil, they represent the 4^th^ leading cause of death and hospitalization due to accidents among children and adolescents up to 14 years of age^14^.

Different products have been used to improve the tissue repair process^4^. There are more than 2,000 types of coverings available on the international market for the treatment of wounds and burns^29^. These can be classified as synthetic (derived from products manufactured or developed in the laboratory) or biological (derived from natural tissues)^23^.

Since 1980, the cellulose membrane obtained by *Gluconacetobacter xylinus* has been used as a temporary substitute for human skin in superficial and deep burns, graft donor areas, mechanical or laser dermabrasions, and venous and arterial ulcers^20^. Some advantages of the cellulose membrane include the fact that they are not toxic or carcinogenic and their biocompatibility, in addition to retaining moisture and favoring granulation at the wound site^21^. However, their manufacture is a low-yield and high-cost process, since it is a bacterial synthesis.

In view of this, the development of a cellulose membrane from vegetables, such as pine wood, would allow the reuse of by-products from the agroindustry and could reduce production costs, favoring sustainability.

Thus, the objectives of this research were to verify the clinical safety and effectiveness of the vegetable cellulose membrane on the healing of 3^rd^ degree burns on the skin of rats and to compare them with the safety and effectiveness of the commercial bacterial cellulose membrane (Membracel^®^)**.**

## METHOD

The study was carried out on the premises of the Experimental Surgery Laboratory of the Medical Research Institute of Mackenzie Evangelical School of Medicine - Paraná (FEMPAR), Curitiba, PR, Brazil. It was approved by the FEMPAR Animal Use Ethics Committee under number 872/2019, governed by Law 11,794.

### Production of cellulose nanofibers

The nanofibers from pine (*Pinus sp*) were produced at the Wood Technology Laboratory of the Brazilian Agricultural Research Corporation (EMBRAPA Florestas), located in Colombo, PR, Brazil.

**FIGURE 1:**
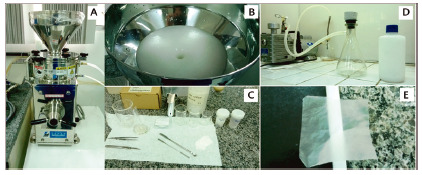
Stages in the production of vegetable cellulose nanofibrils: A) Mass Colloider Masuko mill; B) cellulose suspension being processed by the mill; C) utensils used to produce nanofibrilated cellulose suspension; D) vacuum filtration for membrane production; E) translucent produced film.

The hemicellulose extraction process was carried out by bleaching the cellulosic pulp, obtaining a suspension of nanofibrils with 86% cellulose and 14% hemicellulose. This bleached pulp was dispersed in distilled water and homogenized in a laboratory blender to obtain a “fluffy” paste. For the defibrillation process, this paste at a concentration of 3% on a dry basis, was inserted into the Super Masscoloider Masuko Snagyo mill, under rotation of 1,500 rpm and 20 passes, with the pass being a paste grinding cycle. The membranes were produced by means of filtration in 22 µm nylon screens. The masses of the suspensions necessary to obtain each film were diluted in distilled water to a concentration of 3x10-3g/ml and stirred for complete homogenization. Then, the excess water was removed by compressing the film in glass plates and drying for 24 h in an oven at 60º C. The films were produced with the objective weight of 40 g/m² and diameter of 100 mm (Figure 1). Small perforations were made in the cellulose films with an 18G gauge needle. Before being applied to the wounds of the animals, the membranes were sterilized in an autoclave at 121° C for 30 min, which, under these conditions, the cellulose film does not undergo changes in physical properties^7^.

### Animals used and general care

Thirty-three adult male Wistar rats (*Rattus Norvegicus albinus*), 200 days old and weighing between 450-500 g, were studied. Initially, they remained for an adaptation period and health status observation of 20 days, in order to guarantee the absence of diseases. They received light access to water and standard feed. They were kept at a controlled room temperature between 18-22^o^ C, in the proper humidity conditions of the environment and cyclical control of light and dark every 12 h. They remained in individual polypropylene cages 41x34x18 cm^3^, lined with wood shavings and labeled for identification.

### Experimental design

All were weighed on a Brasmed^®^ digital scale and randomly distributed into three groups (n=11): group 1 - control, with third degree burn, surgical debridement and dressing only with gauze; group 2 - bacterial cellulose membrane, with third degree burn, surgical debridement and application of the bacterial cellulose membrane (Membracel^®^); group 3 - vegetable cellulose membrane, third degree burn, surgical debridement and application of the vegetable cellulose membrane.

### Anesthesia

All remained food fasting for 6 h and water for 2 h. An intraperitoneal injection was performed with ketamine hydrochloride at a dose of 90 mg/kg, associated with xylazine hydrochloride at a dose of 10 mg/kg. During the entire anesthetic period, heart and respiratory rates were observed, in addition to the voluntary movement of the rats, in order to detect complications or manifestations of pain to supplement anesthesia with new doses. The animals were considered anesthetized and ready for the procedure when they lost eye and caudal reflexes.

### Burn induction

**FIGURE 2:**
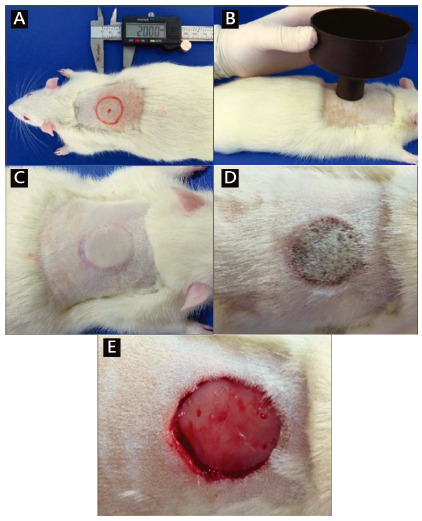
A) Marking of the burn area: circular area in red representing a standardized place for the induction of the burn in all animals; B) positioning of the scalding device; C) aspect of the lesion immediately after the induction of the burn: observe an area of pale skin, surrounded by a hyperemic halo; D) devitalized area with necrotic tissue two days later; E) live wound resulting from surgical debridement

After anesthesia, they were positioned in prone position and immobilized on a surgical board to perform trichotomy in the dorsal region, with an electric epilator. The region to be trichotomized was demarcated 7 cm from the animal’s nasal tip, in the craniocaudal direction (Figure 2A) and from this point, performed in an area of 5x3 cm^2^.

The burn induction site was previously marked with a Sharpie^®^ pen, using a 2 cm diameter plastic circular mold that was positioned in the center of the trichotomized area (Figure 2A). After marking, antisepsis was performed with iodopolividone 10 solution followed by 2% alcoholic iodine solution.

**FIGURE 3:**
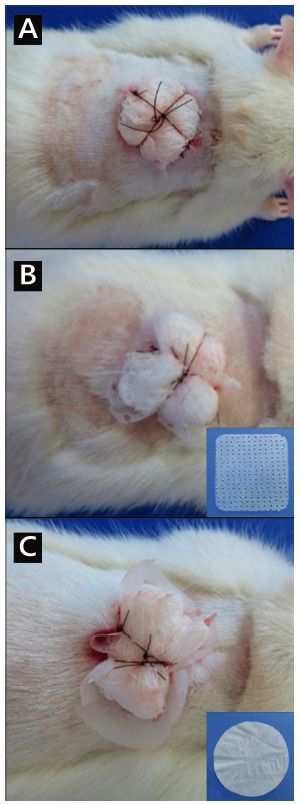
Procedures protected with a secondary Brown dressing (note the yellow “X” appearance of the knots, after dressing): A) only gauze (group 1 - control); B) Membracel^®^ (group 2); C) vegetable cellulose membrane (group 3)

Burns were performed by scalding. Inside a beaker container, the water was heated to 98,4º C, measured with a digital thermometer. In order to define the scald, a polypropylene funnel device was used. The device was pressed on the animal’s back, leaving the smaller opening, 2 cm in diameter, in contact with the skin (Figure 2B). The funnel was then filled with 20 ml of boiling water; 30 s were recorded with a digital timer and, afterwards, the device was removed, taking care that the water did not flow to other parts of the body. The form of induction of the burn was the same in all animals, obtaining circular lesions with an area of ​​3,14 cm² (Figure 3C). After the induction of burns, the lesions were kept open in the animals of the three groups, allowing visualization of the evolution of the wound.

### Debridement of wounds and application of dressings

On the second day of the burn, after the new anesthetic induction (described above), surgical debridement of the wound was performed by excising the necrotic area with a 15 Feather^®^ scalpel blade (Figures 2D and 2E). After excision of the devitalized tissue, the dressings and membranes previously designated for each group were applied.

In order to avoid the removal of the membranes by attempting the animals’ paws and mouths, a secondary dressing was chosen, using Brown’s technique. Four simple equidistant stitches (4-0 nylon) were made at the edge of the lesion. The wires were kept 6 cm long. Folded gauze was placed under the lesion and then knots were made between two opposite threads, forming an “X” (Figure 3A).

In group 1, the wound bed was covered only with gauze and later, Brown’s dressing was made (Figure 3A).

In group 2, bacterial membranes (Membracel^®^, Figure 3B) were applied, leaving an excess of 0.5 cm in relation to the size of the wound. Then, Brown’s dressing was performed (Figure 3B).

In group 3, vegetable cellulose membranes (Figure 3C) were applied, with an excess of 0.5 cm in relation to the wound size. Then, the Brown dressing was applied (Figure 3C).

The animals were followed up daily for 15 days to check systemic clinical parameters and local aspects of the wound.

On the third day after applying the membranes, Brown’s bandages were removed and the wounds were cleaned with iodopolividone solution.

### Clinical care

After the surgical procedures, the animals were observed during anesthetic recovery, being kept warm in thermal plates. Analgesia was performed by administering 5 mg/kg of tramadol hydrochloride every 12 h for the first five days after the burn and debridement.

### Parameters evaluated after the procedures

All animals were evaluated daily, in order to detect local and systemic changes resulting from the procedure. The clinical parameters evaluated were: general status (movement, alertness and responsiveness to the environment); appetite; weight variation during the follow-up period; and mortality.

### Macroscopic aspects

To assess signs of allergic reaction, inflammation, infection or other local complications, the wounds were examined daily. The macroscopic aspects evaluated were: bleeding; phlogistic signs (flushing, heat, pain, edema); exudate; abscess formation; and presence of necrotic tissue (crusts).

In addition to these parameters, on the last day of the survey (15^th^ day), each animal’s lesion was measured with the aid of a Zaas Precision^®^ digital caliper. The measurements of the diameter of the lesions were obtained for later calculation of its final area.

From the wound area, the percentage of injury contraction was calculated, as proposed by Agren et al^2^, in which the percentage of contraction (CP) consists of the result of the final area (FA) minus the initial area (IA), divided by the initial area and multiplied by 100.

$$\;Contraction\;percentage\;(CP)\;=\frac{\;Final\;Area\;(FA)\;-\;Initial\;Area\;(IA)\left(mm^{2}\right)}{\;Initial\;Area\;(IA)\left(mm^{2}\right)}\times 100(\%)$$

### Microscopic analysis

On the 15^th^ day after the burn, the rats were euthanized by anesthetic overdose. Then, a transverse fragment of the lesion, in the craniocaudal direction, was removed for histological analysis. The specimens were placed in vials containing 10% formalin for 48 h. After that period, the fragments were sent to the Experimental Pathology Laboratory of Mackenzie Evangelical School of Medicine. The slides were prepared with 4 µm cuts and stained with H&E and picrosirius red.

For histological analysis, the degree of healing (1, 2, 3 and 4) and the percentage of type I and type III collagen were considered. For these evaluations, the images of the slides stained with H&E were obtained using an Olympus BX 50 multi-head optical photomicroscope and analyzed using the Axiovision software (Carl Zeiss^®^).

In the analysis of the degree of healing, the comparative table by Greenhalgh et al^10^ was used, which classifies each degree according to the intensity of granulation tissue, the amount of inflammatory cells and fibroblasts, as well as neovascularization and epithelialization.

The slides stained with picrosirius red were observed under an optical microscope under polarized light, to determine the concentration of type I and type III collagen fibers in the area corresponding to the scar.

The digitalized images of the scars visualized with the polarizing lens were submitted to the Image Pro Plus 4.5^®^ program, which recognized reddish colored regions and thick fibers (collagen type I - mature) and greenish color and fine fibers (collagen type III - immature), calculating the concentration of these collagen subtypes in the studied area.

### Statistical analysis

The results of the quantitative variables were described as mean±standard deviation from the mean. Categorical variables were described by frequency and percentage. For the comparison between groups in relation to quantitative variables, the one-way analysis of variance model (ANOVA) and the LSD test (least significant difference) were used for multiple post-hoc comparisons. For the analysis of final weight and weight variation, the covariance analysis model (ANCOVA) was used, adjusting for the initial weight. Categorical variables were analyzed considering the chi-square test and the adjustment of logistic regression models. Values ​​of p<0.05 were considered significant. The data were analyzed using the computer program Stata/SE v.14.1. StataCorpLP, USA.

## RESULTS

### Clinical evaluation

The animals remained clinically well and active throughout the experiment, showing normal appetite, behavior, feces and urine. Anesthesia and surgical procedures evolved without major complications. A rat in the control group died on the first day of the study, during anesthetic induction and was not replaced in the experiment.

### Weight variation

The mean values of the weight variation of the animals in the three groups showed that there was no statistical difference in the weight variation between the groups (p=0.952) in the studied period.

### Macroscopic evaluation

**FIGURE 4:**
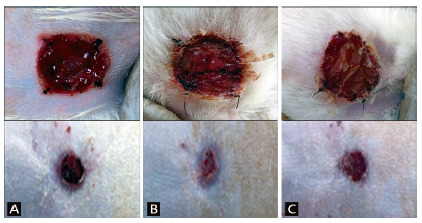
Appearance of the lesions after removing the secondary dressings (above) and at the end of the research (below): A) group 1 control; B) group 2; C) group 3. It is noted above that in both groups 2 and 3 there was protection of the wound bed and there was no bleeding as seen in A.

Brown’s secondary dressings were removed in the animals of the three groups. In groups 2 and 3 (cellulose membranes), the removal did not present any difficulties and there was no bleeding. It was observed that the vegetal and bacterial membranes were found in the lesion bed, preventing the gauze from the secondary dressing to adhere to the wound and cause bleeding. In the animals in the control group, the gauze was directly adhered to the lesion bed, causing bleeding and subsequent crusting in six animals (60%, Figure 4).

Throughout the research, no signs of infection or abscess formation were observed in the wounds of the animals in the three groups. At the end of the study, 15 days after the burns, all membranes had detached themselves from the lesion and signs of their presence were no longer observed in groups 2 and 3. The wounds of the three groups remained red and covered with granulation tissue (Figure 4).

### Scar contraction

Table 1 shows the values ​​of the final area of ​​the wounds, absolute scar contraction, as well as the rate of scar contraction for each group, after the follow-up period.

At the end of the research, the lesions treated with the plant and bacterial membranes evolved with the highest contraction rates, presenting wounds with smaller areas than the control group.

Comparing the measurements of the final area of ​​the wounds, absolute scar contraction and contraction rate, there was no statistical difference between the groups (p˃0.05).

**TABLE 1:** Values ​​of the wound area (mm2), absolute scar contraction (mm2) and percentage of scar contraction

Variables	Group	Mean±standard deviation	p*
Area	1	40.0±16.7	
	2	28.8±14.4	0.447
	3	34.7±26.4	
Contraction (IA (314 mm) - FA)	1	274.0±16.7	
	2	285.2±14.4	0.346
	3	275.1±24.9	
Contraction rate	1	87.3±5.3	
	2	90.8±4.6	0.347
	3	87.6±7.9	

*ANOVA, p<0,05; IA - initial area; FA -final area

### Microscopic analysis

#### Degrees of healing

**FIGURE 5:**
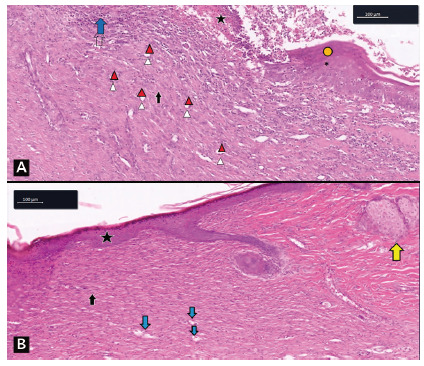
Histological section photomicrograph stained in H&E - healing grade III: A) group 3 animal - inflammatory cells and mixed infiltrate with lymphocytes and neutrophils (blue arrow); blood vessels (red arrowheads); fibroblast (black arrow); regenerating epithelium (yellow circle); presence of crust (star) (40 x); B) healing grade IV animal of the control group - scar tissue on the left, with vessels and more mature fibrocollagenous tissue, rare inflammatory cells and covered by the epidermis; uninjured tissue on the right, more eosinophilic, less cellular and with skin attachments; sebaceous gland (yellow arrow); epithelium covering granulation tissue (star); blood vessels (blue arrows); fibroblasts (black arrow) (40 x)

In all groups, an advanced degree of healing was observed. The wounds were partially or totally epithelialized, filled with thick granulation tissue and neovascularization. In animals in groups 2 and 3, more than 60% of the wounds were in grade III healing (Figure 5A). In the animals of the control group (5B), there was a predominance of wounds in grade IV healing.

Table 2 shows the frequencies and percentages of degrees of healing (I to IV) obtained in animals in groups 1, 2 and 3.

### Percentage of types I and III collagen

The percentages of type I and III collagen obtained in each group are listed in Table 3. The proportion of type I and type III collagen was homogeneous in the three groups, with moderate to abundant collagenization, predominantly of mature collagen. Note: The percentages of collagen I and III add up to 100%, so the p values of the statistical tests are the same.

**TABLE 2:** Absolute and relative frequency of degrees of healing in groups 1, 2 and 3 after the follow-up period

Degree of healing	Group		
	Control	Membracel^®^	Vegetal membrane
I	0	0	0
	(0%)	(0%)	(0%)
II	2	0	2
	(20.0%)	(0.0%)	(18.2%)
III	1	8	7
	(10.0%)	(72.7%)	(63.6%)
IV	7	3	2
	(70.0%)	(27.3%)	(18.2%)

Grade 1 - Absence of granulation tissue; grade 2 - scarce and immature granulation tissue, minimal epithelialization; grade 3 - medium granulation tissue, neovascularization, moderate epithelialization; grade 4 - abundant and well vascularized granulation tissue, abundant epithelialization.

**TABLE 3:** Percentage of type I and III collagen in the wounds of animals in groups 1, 2 and 3 at the end of the study

Variables	Group	Mean±standard deviation	p*
Collagen I	1	72.3 ± 16.0	0.135
	2	78.6 ± 11.7	
	3	68.0 ± 15.5	
Collagen III	1	27.7 ± 16.0	0.135
	2	21.4 ± 11.7	
	3	31.9 ± 15.5	

* Kruskal-Wallis non-parametric test, p<0.05

## DISCUSSION

### Sample

The Wistar rat was chosen for the study because it is an animal of easy experimental manipulation. In addition to great resistance to infections and the well-known anatomy and physiology, it allows standardization regarding age, weight, sex and diet. In a 2014 study, Abdullahi et al^1^ explained the need to use animal models to clarify the pathophysiological mechanisms of burns and to assess the effect of new therapies. Thus, the present research tested a cellulose membrane of plant origin to verify its influence on the healing process of burns in animals. Due to the accelerated metabolism, the healing phases are shorter in rats when compared to humans, which allows researchers to obtain results more quickly in studies involving healing processes^1^. Other overriding advantages for choosing this animal were small size (less amount of membrane used), reduced cost and availability.

The main disadvantage of using them as experimental models are the anatomical differences with human skin, such as the distribution of hair follicles and the presence of the fleshy panicle muscle in rodents. This muscle leads to healing basically by contraction, different from the epithelialization that predominates in humans^6^. In addition, there is no firm adherence of the rats’ skin to the underlying structures^1^.

According to Coelho et al^7^, approximately 80% of medical research uses rodents as experimental animals and in the case of experimental research involving burns and scarring, this is one of the models that gives better results.

### Burn induction

Experimental models are essential for testing therapies before introducing them to clinical use. When evaluating new treatments for burns, it is important that the model used allows the creation of uniform and reproducible injuries^28^.

The techniques used to generate burns in experimental models include heated liquids, incandescent instruments and electricity^15^. In the present study, scalding with boiling water was performed, which represents the main cause of burns in children^12^.

In a 2012 systematic review, Mitsunaga Junior et al^15^ showed that 55.1% of the studies performed trichotomy before the burns. Similar to these studies, the present research also performed trichotomy. In 2014, Cai et al^6^ emphasized that the phase in which hair development is found (catagen and telogen) can influence the depth of the acquired lesion.

The back of the rat was the defined place to perform the burn, allowing the standardization of the healing process. As reported by Cai et al^6^, wounds located in different areas of the body heal in different ways. In addition, the wound is more protected from further trauma on the back, as it is difficult to access through the animal’s mouth and paws^15^.

Fantinati et al^8^ used a plastic cylinder device resistant to high temperatures and exposed the animal to hot liquid to obtain 3^rd^ degree burns on the back of the murines. Coelho et al^7^, carried out deep 2^nd^ degree burns, depositing boiling water in a cut 20 ml syringe, which was kept in contact with the rat’s skin for 20 s. Similar to these studies, the present research also used a device to perform the burn, but in the shape of a funnel, made of material resistant to high temperatures. This device proved to be more practical and safer than the syringe for placing boiling water in the device, reducing the risk of the liquid running and burning the researcher’s hand.

To obtain 3^rd^ degree lesions, the water was heated to 98° C and remained in contact with the skin for 30 s. The region exposed to boiling water showed a pearly white appearance, a macroscopic characteristic of full-thickness burns. The present model proved to be practical and effective for inducing 3^rd^ degree burns on the skin of rats, making reproduction possible.

### Surgical treatment of burns

#### Debridement

According to Phelan et al^18^, early excision of necrotic tissue and wound closure are one of the greatest advances in the treatment of severe burns. Excision reduces bacteremia, the production of endotoxins and the release of inflammatory mediators^17^. This reduces the chances of sepsis and multiple organ failure, the main causes of death in severe burns. The most appropriate period of excision is still controversial. In the present study, it was decided to perform it 48 h after the thermal injury, corroborating a study by Phelan et al^18^ who mentions that ideally, the excision should be performed within 24-72 h after the burn.

After excision, skin grafting is performed simultaneously, if available. In some cases, temporary dressings or other alternatives (skin substitutes) are necessary until definitive reconstruction is performed^18^. The purpose of this study was to investigate the effectiveness of plant cellulose membranes as natural biological dressings for the treatment of 3^rd^ degree burns and to verify their effect on tissue repair.

#### Cellulose membranes

Studies on the effect of cellulose membranes in the treatment of burns have increased in recent years, mainly on 2^nd^ degree injuries. Muangman et al^16^ presented the contribution of bacterial membranes to the healing process of 2^nd^ degree burns on the face. Coelho et al^7^ carried out a study with a vegetal membrane in 2^nd^ degree deep lesions in rats and obtained results similar to those of the bacterial membrane commercialized in Brazil (Membracel^®^).

Studies involving the use of plant cellulose in healing are still scarce. This is one of the first studies to evaluate the action of plant nanofibers, originating from pine, in the tissue healing process, being the first study in the literature to use this product in the treatment of 3^rd^ degree burns.

One of the main advantages of bacterial membranes is their ability to adhere and adapt to the wound bed, including the face and mobility sites^9^. In addition, its transparency facilitates the daily assessment of injuries^19^. Studies have shown that these characteristics have also been maintained in pine membranes. Membracel^®^ has pores with position memory, which do not change in diameter over time and allow the free passage of excess secretions^29^. As a result, perforations were made in the studied membrane, creating artificial pores for the passage of exudate. However, it is a product undergoing testing and not yet commercialized. Thus, more research is needed before standardizing these aspects of the membrane, such as pore thickness, size and shape.

### Clinical evaluation

After application of the membranes, no local and systemic complications were observed in the animals. The weight gain was uniform in all groups and within the expected for the species, demonstrating that the appetite remained adequate and that the studied membrane did not cause discomfort or worsening of the general condition. This suggests the clinical safety of the tested products. These data are compatible with Hakkarainen et al^11^ and Coelho et al^7^ who tested cellulose membranes of plant origin in wounds on the backs of rats and proved the biocompatibility and atoxicity of the material.

The membranes were covered with a secondary dressing, made with gauze fixed with nylon stitches intertwined in the skin - Brown dressing - to prevent the rats from removing the membranes, causing additional trauma with the mouth and paws, and that the box shavings adhere to the wound. The present study was similar to the one carried out by Ramalho et al^25^, who successfully used this type of dressing to cover and protect skin grafts on the back of rats.

No infections and inflammations were observed with the use of Brown’s dressing in the present study.

### Macroscopic evaluation

Although without statistical difference, the analysis of these measures allowed to verify the healing speed, which showed to be faster in groups 2 and 3, and also to calculate the final burn area. The average of the final areas of the groups treated with cellulose membranes was lower than in the control group. The rate of contraction of the lesions in groups 2 and 3 were higher than in the control, revealing faster healing in these groups. These results corroborate those found by Hakkarainen et al^11^, who observed faster healing with the use of cellulose membranes in graft donor areas, when compared to another dressing used in the treatment of burns. Coelho et al^7^ states that the cellulose membrane works as a means of growth and adhesion of epithelial cells, favoring their migration to the center of the lesion and, consequently, the wound closure.

In the present study, the animals did not show signs of local sensitivity, such as edema, flushing, pain or itching after the application of the plant membrane. These results are also compatible with those found by Coelho et al^7^. BrassolattI et al^5^ explained that bacterial cellulose biomaterials decrease the inflammatory response, when compared to conventional grafts in total skin wounds of rats. Muangman et al^16^ applied the bacterial dressing to a 54-year-old patient with a superficial burn and did not observe signs of skin irritation or allergic reactions. In the case of plant membranes, Hakkarainen et al^11^ performed in vivo tests on rats and, after proving the safety of the material, performed clinical studies in graft donor areas of nine patients and no adverse effects were found.

During the manipulation of the lesions, there was greater difficulty in removing the gauze dressing from the animals in the control group, since it was attached to the wound bed, causing bleeding and crusting. These data corroborate the study by Rogers et al^27^, which showed that the gauzes favor local dryness and cause strong adherence to the lesion bed, causing pain when removed, in addition to removing newly formed scar tissues. Traditionally, the gauzes are used as material for dressing due to its high permeability, absorbing fluids secreted by the lesion^11^.

On the other hand, Barud et al^3^ explain that the membranes adhere well to the wound bed and favor the maintenance of local humidity, forming a barrier between the lesion and the environment. This allows the exchange of secondary dressings with less difficulty and pain. Similarly, in animals in groups 2 and 3 (with membranes), the secondary dressings were also removed easily, without bleeding and without removing the granulation tissue.

There were no signs of infection, purulent exudate or abscess formation in the lesions of the groups treated with both cellulose membranes. However, it cannot be said that the membrane prevented infections, since there were no cultures of microorganisms. Powell et al^22^ conducted an in vitro study that demonstrated that the plant cellulose membrane was effective in inhibiting the growth of *Pseudomonas aeruginosa*. However, Hakkarainen et al^11^ reported that bacterial suspensions of *S. aureus* and *P. aeruginosa* did not have their growths affected by vegetable cellulose, suggesting that the material itself does not have antibacterial properties. Rajwade et al^24^ report that the membrane acts as a physical barrier between the wound and the environment and, therefore, prevents the occurrence of infections.

### Microscopic evaluation

In the H&E analysis, there was a predominance of tissue rich in fibroblasts and collagen, as well as neovascularization and epithelium in regeneration or neoformed in the animals of the three groups. In all animals in the group treated with Membracel^®^, the wounds were in advanced stages of the healing process (grades III and IV), also observed by Lin et al^13^. In groups 1 and 3, most of the lesions were also in advanced degrees, but approximately 20% were still in degree II of healing.

Picrosirius red staining was used to verify the area and density of total collagen, in addition to the differentiation of young and mature fibers. The healthy dermis contains approximately 80% of type I collagen (mature) and 20% of type III (immature); granulation tissue, on the other hand, expresses 30-40% of type III collagen^26^.

In the present study, no statistical difference was observed in the proportion of collagens between the three groups, similar to the research by Brassolatti et al^5^, who also did not find differences in the percentage of collagen fibers in groups treated with cellulose membranes in relation to the control. Unlike what was reported by Yaguishita et al^29^, who observed a higher proportion of mature collagen in the animals treated with Membracel^®^ compared to animals in the control group, this difference may have occurred depending on the type of lesion evaluated. While the present study evaluated burns, Yaguishita et al^29^ produced wounds by removing a skin fragment with a scalpel, which may have influenced the healing process. In addition, they evaluated the groups weekly, at 7, 14, 21 and 28 days, while in the present study, microscopic evaluation was performed only on the 15^th^ day.

Although no statistical difference was found in the microscopic analysis between the three groups, in macroscopic analysis, cellulose membranes were more effective in minimizing the formation of crusts, pain and bleeding during dressing changes. Due to the fast metabolism of the rats, at 15 days of follow-up, the wounds of the three groups were similar in size. However, it is possible to observe faster and more uniform closure in wounds treated with cellulose membranes, as already observed in several other studies^7^ ^,^ ^11^ ^,^ ^29^.

### Future perspectives

Research involving nanotechnology and nanomaterials has introduced new therapeutic horizons in the medical field, producing bioactive materials for the treatment of wounds.

Dressings produced from plant nanocellulose have shown similar efficacy to bacterial cellulose in several studies. The lower production cost of vegetable cellulose, combined with the greater availability of raw material, make it possible to produce it on a large scale, allowing the availability of this resource for a greater number of patients.

The results of this study contribute to improving knowledge about plant cellulose nanofibers as a therapeutic alternative in the treatment of deep burns.

Further research is needed to consolidate the results obtained and verify other aspects relevant to the use of the plant cellulose membrane, such as antibacterial action and systemic inflammatory response.

## CONCLUSIONS

Both cellulose membranes - vegetable and bacterial - are: 1) clinically safe for the treatment of 3^rd^ degree burns in rats; 2) the macroscopic evolution of the wounds was similar between the groups treated with them, being superior to the control group; 3) microscopic evaluation showed no significant difference between the groups treated with bacterial and vegetal cellulose membranes in relation to the control.
